# Magicmol: a light-weighted pipeline for drug-like molecule evolution and quick chemical space exploration

**DOI:** 10.1186/s12859-023-05286-0

**Published:** 2023-04-26

**Authors:** Lin Chen, Qing Shen, Jungang Lou

**Affiliations:** 1 Yangtze Delta Region (Huzhou) Institute of Intelligent Transportation, Huzhou University, Huzhou, China; 2 School of Electronic Information, Huzhou College, Huzhou, China; 3 Zhejiang Province Key Laboratory of Smart Management and Application of Modern Agricultural Resources, School of Information Engineering, Huzhou University, Huzhou, China

**Keywords:** Generative models, Reinforcement learning, Deep learning, Synthetic accessibility, De novo drug design

## Abstract

The flourishment of machine learning and deep learning methods has boosted the development of cheminformatics, especially regarding the application of drug discovery and new material exploration. Lower time and space expenses make it possible for scientists to search the enormous chemical space. Recently, some work combined reinforcement learning strategies with recurrent neural network (RNN)-based models to optimize the property of generated small molecules, which notably improved a batch of critical factors for these candidates. However, a common problem among these RNN-based methods is that several generated molecules have difficulty in synthesizing despite owning higher desired properties such as binding affinity. However, RNN-based framework better reproduces the molecule distribution among the training set than other categories of models during molecule exploration tasks. Thus, to optimize the whole exploration process and make it contribute to the optimization of specified molecules, we devised a light-weighted pipeline called Magicmol; this pipeline has a re-mastered RNN network and utilize SELFIES presentation instead of SMILES. Our backbone model achieved extraordinary performance while reducing the training cost; moreover, we devised reward truncate strategies to eliminate the model collapse problem. Additionally, adopting SELFIES presentation made it possible to combine STONED-SELFIES as a post-processing procedure for specified molecule optimization and quick chemical space exploration.

## Introduction

Generative models, which use computational methods to devise molecules inversely, can be roughly separated into several categories: variational autoencoders (VAEs) [1–3], generative adversarial networks (GANs) [4, 5], recurrent neural networks (RNNs) [6, 7], and flow-based models [8, 9]. In essence, a generative model learns valid molecule presentations [10] from an extensive, cleaned database. For RNN-based models, while training, the input is like a “prefix” [11]; for each iteration, a particular prefix is fed into the model, and the next character is defined as the training target. Concerning the existence of the hidden layer, which the ability of RNN models to process sequential data, RNN models take the output of last step as the next input and thus memorize coherent sequential information. During this process, an initial character and a terminal character are used to indicate the start and termination of generation. Following the established probabilistic rules, a well-trained generative model can “reproduce” the process while generating molecules with different sampling strategies (different sampling temperatures [12], etc.), which accounts for the validity and novelty of generated molecules.

Many experiments [13–15] have confirmed the feasibility and generative capacity of RNN-based models. Recently, Alan et al. [16] compared chemical language models and concluded that RNN-based models prevail over VAE-based models when reproducing the molecule distribution of the training set. However, biases of the training data can lead to corresponding tasks to be grossly overestimated [17]; Without extra optimizations, RNN-based models perform well in terms of common evaluate metrics (for instance, novelty, validity, and originality), but many generated molecules contain unwanted structures, or they are just not available because difficult to synthesize.

We see this problem is caused by the partiality of the evaluation metrics, especially the metric - novelty. Specifically speaking, the novelty of a generative model is defined as:

1 $$\begin{aligned} \begin{aligned} Novelty = 1 -\frac{\left| {\text {set}}\left( V_{m}\right) \cap N\right| }{\left| {\text {set}}\left( V_{m}\right) \right| } \end{aligned} \end{aligned}$$

where ($V_{m}$) is a batch of valid, non-duplicate generated molecules, and *N* is the original training set. Any molecules that have never emerged in the training set will contribute to the novelty score. Concerning the aforementioned situation, the classic novelty evaluation metric cannot reflect such implicit structural problem, and it should be regard as “permissive novelty” [18].

Meanwhile, Deep generative models (DGMs) are not the only way to conduct efficient chemical space exploration. Recently, some research utilized genomic algorithms (GAs), such as Monte-Carlo Tree Search (MCTS), instead of DGM, demonstrating that GAs served as potent candidates for searching for desired chemical compounds [19, 20]. These search-based methods generally regard molecule fragments as tree nodes, and the whole process can be viewed as searching for a feasible connection between the existing root and leaves [21, 22]. Feasible connections not only ensure the validity of generated molecules but also make the molecular exploring process more efficient. However, the search process needs to get feedback from third-part supervision, which could be a scoring function, a neural network [22], or some mechanism such as expectation maximization [23]. This step requires extra training and must be devised precisely to ensure it leads the search process in the right direction.

In this paper, we attempted a new pattern of combining GA and DGM and treat novel molecule exploration as a two-step task. First, we re-mastered a three-layer stacked RNN model as our backbone model for quick chemical space exploration. Then we turn to solve the problem of permissive novelty among RNN-based models using reinforcement learning and the target is reinforcement score (for details, see chapter Reinforcement Score). During this, we adopt SELFIES [24] as the molecule presentation instead of SMILES [25]. Second, when the network converged, we utilize its exploration power to find the evolution target (a molecule with ideal properties), and the target will lead the optimization for our specified molecule.

The main contribution of this paper are summarized below: (1) We re-trained a more efficient backbone model without inheriting the former framework and the post-processing bases on reinforcement score could make our model cater to different requirements. (2) We devised a light-weighted baseline that combines GA and DGM for specified molecule evolution without introducing extra parameters,the whole process is intervention-free and does not need further supervision. (3) We issued reward truncate strategies to reduce the side-effects of reinforcement learning optimization and prevent model collapse, which could to be transferable to other tasks.

## Methods

### Molecule presentation

To make the model learn contextual relation and presentation of valid chemical compounds, a certain chemical molecule must be first presented as a meaningful vector [26]. As formerly mentioned, our baseline would be combined with STONED-SELFIES [27] for molecule exploration, and therefore we used SELFIES, which is a novel and robust molecule presentation. SELFIES mitigated the problem of the random invalidity found with SMILES and ensured validity after structure modification.

We obtained excellent experimental results when adopting SELFIES for encoding molecules as network inputs (Table 1), which shows superiority not only in the validity of generated molecules but also reduced training cost.

### Dataset and processing

A larger dataset provides more abundant combination of molecule fragments, which empowers DGM to search the enormous chemical space. Therefore, we use the ChEMBL30 dataset (https://www.ebi.ac.uk/chembl/) [28], which contains more than 2.2 million compounds and 1.92 million small molecules. We derive all small molecules for data preprocessing. The data cleaning procedure is done by Rdkit (https://www.rdkit.org/) [29] as follows: (1) confirm the validity of SMILES in the original dataset; (2) use *LargestFragmentChooser* to select the largest fragment while including more than one fragment; (3) use *uncharge* to clean the electrons and make them neutral; (4) (optional) use *TautomerEnumerator* to handle the tautomerism factors; and (5) convert all valid SMILES into canonical form, and we arbitrarily ignored isomeric SMILES.

**Fig. 1:**
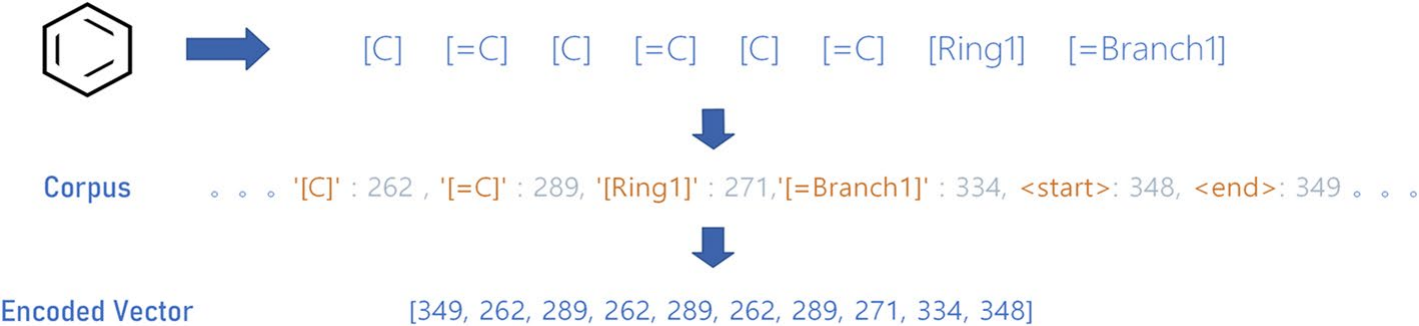
The encoding process and an example of a benzene molecule, which is first decomposed into items and structural tokens, and then each part is encoded by the established chemical corpus. Finally, the molecule is transformed into a meaningful vector

After data cleaning, we take the SELFIES package to convert all selected small molecules to SELFIES presentation and make up a chemical corpus of size *c*. This corpus records all chemical substructures, such as $[Branch1\_1]$ or $[C+expl]$, and three placeholders named $<start>$, $<pad>$, and $<end>$, which are the start generation, padding, and end generation, respectively. While training, we first decompose all molecules in our cleaned dataset into small fragments. Every fragment corresponds to one token in *c*, and the process is shown in Fig. 1. We note that not all molecules can be encoded as SELFIES presentation for several reasons, such as violating the predefined rules, we excluded all these molecules. Before we feed the data into our model, molecules are splited and transformed into meaningful vectors according to the chemical corpus. Here we choose the length of the longest encoded molecule (*l*) of each batch as the final vector length. A placeholder pads any molecule that does not match the final length in order to facilitate training. Finally, $<start>$ and $<end>$ are added to the head and tail of the encoded vector, respectively.

### Backbone model

A common problem with DGMs is the chemical invalidity of generated strings while adopting SMILES as the model input; this is usually caused by unmatched parenthesis [30], the emergence of DeepSMILES [31] aims to solve this problem. Because of this, DGMs need more training epochs to reach convergence and get rid of the invalidity problem, for example, ReLeaSE [32] used stacked memory layers to enhance its capacity.

**Fig. 2:**
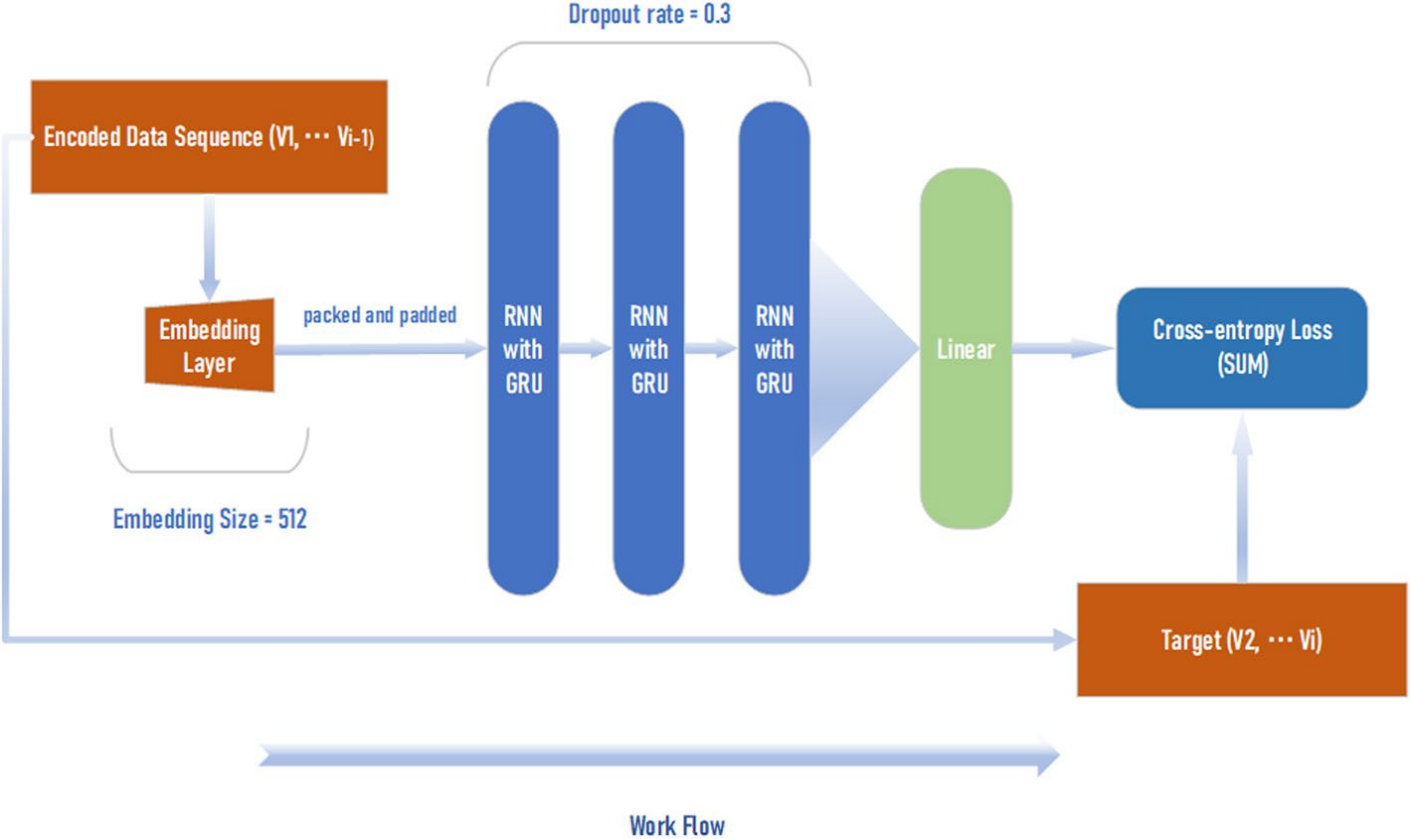
Data flow and backbone generative model for molecule generation

In our work, the molecule presentation has changed, and we define our work as a light-weighted pipeline. Thus, we turn to not use the same generating networks with pre-trained weights, and we instead trained our backbone model. The workflow and model structure is shown in Fig. 2. We adopt a three-layer stacked RNN model with GRU [33]. GRU is an alternative solution to LSTM [34] that can ease vanishing and exploding gradients [12], and thus make it possible to update more effectively during backpropagation. Compared with LSTM, GRU only contains two gates instead of three, thus reducing the training time and network parameters without sacrificing model performance. The two gates of the GRU are the reset gate and update gate. The reset gate controls the information dependency of latest time $h_{t}$ of last time $h_{t-1}$, while the update gate determines the extent of information to be reserved from last time $h_{t-1}$.

Here, we expect our model to learn the valid presentation while also being confined by chemical properties such as chemical valance. Given a sequence of encoded vectors (*V*_*1*_, ..., *V*_*i*_), we let the model predict the distribution of the word (*V*_*i + 1*_). Take a common molecule as an example: if the model receives the sequence '*c1ccccc*', we want the model to learn to maximize the probability distribution of the word* '1'*, and yield the desired molecule. Formally, given a vector *V* we try to maximize the probability of the equation. 

2 $$\begin{aligned} \begin{aligned} -\sum _{i=1}^{o}P(i) log_iP(V_{i+1}| V_1,V_2...V_i) \end{aligned} \end{aligned}$$

where *P* is the probability that each token in *c* is chosen as the next character, and *i* is the time step.

**Fig. 3:**
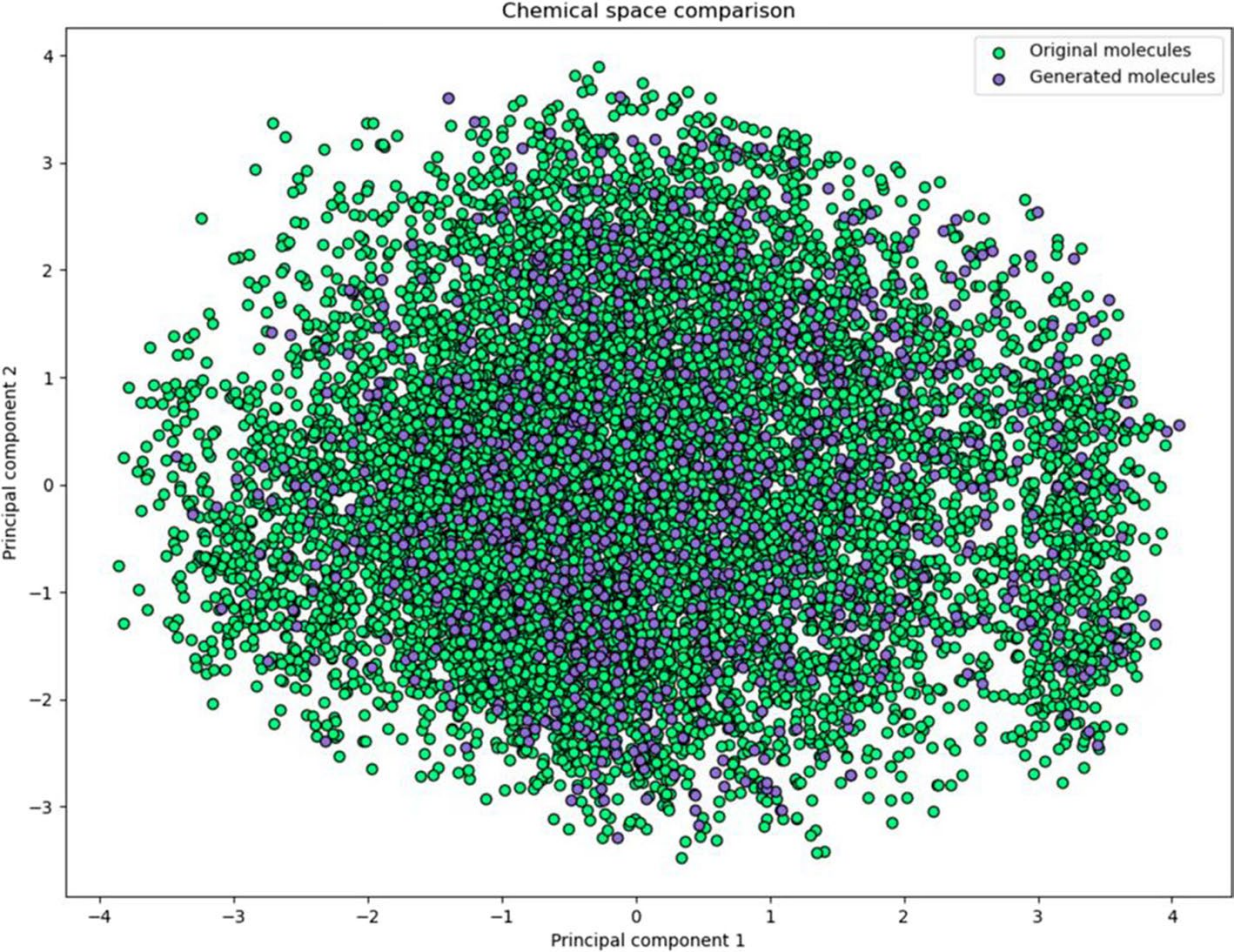
Visualization of training molecules and generated molecules within chemical space using PCA. For convenient viewing, the data is diluted 100 times

After training, we sampled 10,000 molecules to evaluate the generative capacity of our model. We utilize principal component analysis (PCA) [35] and select the first two principal components and visualize them to confirm the exploration capacity of our generative model (Fig. 3). Note that, in our experiment, the whole chemical space is defined as the possible combination derived from corpus *c*, and for SELFIES presentation, the corpus contains 148 different tokens. For both training and sampling, we take all available tokens into consideration, and thus ensure that the whole chemical space is included.

The generation result illustrates that our backbone model reached over 99% validity of generated molecules, diversity, and novelty, as shown in Table 1. These results are discussed in detail in the next session.

### Model optimization

**Fig. 4:**
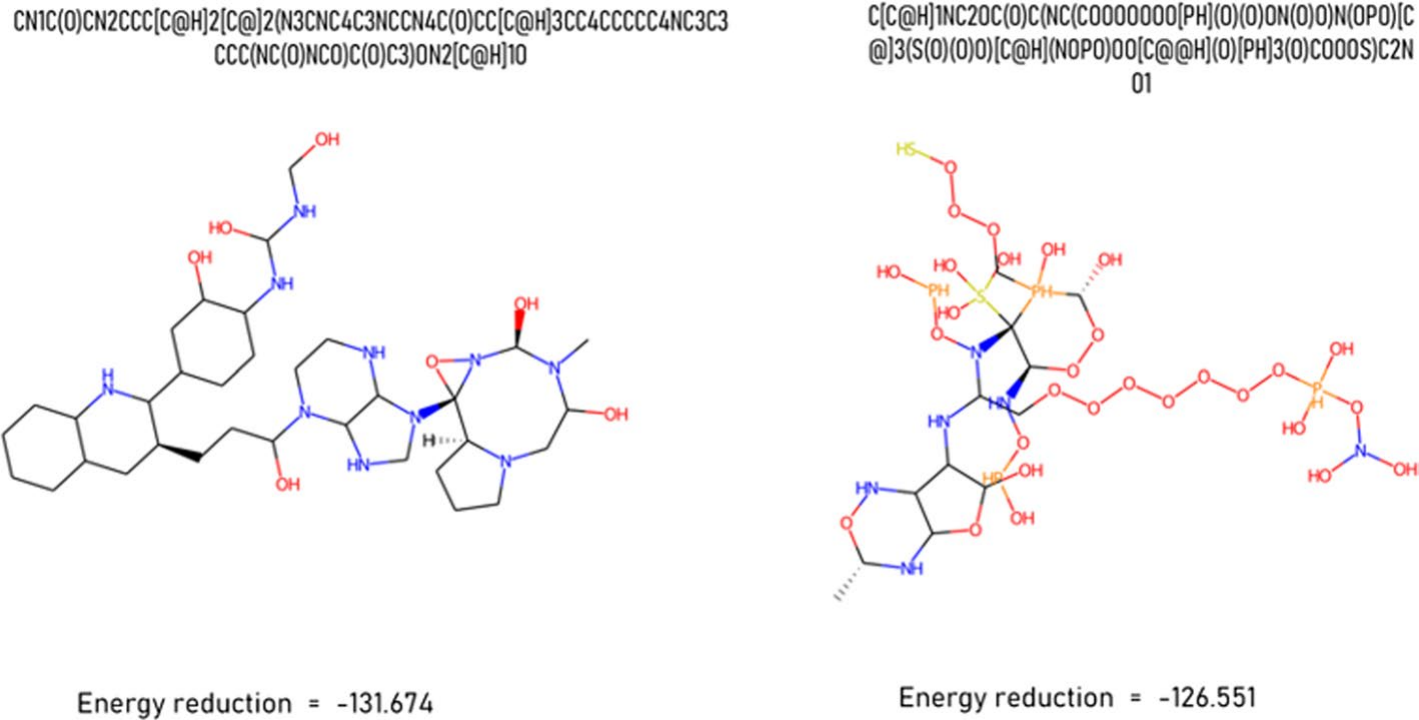
The deep learning model generates these molecules. Both molecules seem to own high binding capacity with the main protease of Covid-19 (code:6LU7). The first molecule is difficult to synthesize because of its structural complexity, while the other has no meaningful chemical structure

As we inferred, RNN-based models perform well in terms of common evaluation metrics, but many generated molecules contain unwanted structures. In Fig. 4, we exhibit two molecules that may cause problems while conducting virtual screening [36] with the goal of seeking drug-like compounds (the binding score is provided by IGEMDOCK [37], and the synthetic score is supplied by SYBA [38]).

In silico molecule design can always be formulated as an optimization problem and it has been widely explored [39, 40]. However, the optimization may be problematic. First, multi-object optimization is still a problem in the drug design field because a certain compound must obey multiple physicochemical properties to be a drug candidate, and a single property being varied may lead to the changing of another property [7]. Second, pursuing too much on some properties may not work well; it is a bit comical that a molecule with the highest LogP would be such a long carbon string and of course is of no means for molecule design [41].

We seek the possibility of reducing the training cost while putting the model ahead and expect it to generates molecules of high quality. Thus, we focus on optimizing only one important property - synthetic accessibility [42, 43], and the following reasons described our opinions: (1) We regard the permissive novelty as a problem caused by lacking a structural constraint. From an economical perspective, the structure of drug-like molecules is often regular and easy to synthesize. Thus, changing synthetic accessibility (SA) may increase the quality of produced molecules. To accomplish this, we utilize SYBA instead of the traditional SA score as our synthesis difficulty judgment. The design of SYBA takes the synthesis routes into concern, which could thus be a good-quality evaluation tool for the generated molecules. (2) The next step of our pipeline may bring structure modification to a certain molecule. Since this modification may contain randomness, the structure of the variants may deteriorate. Thus, we explicitly optimize it to mitigate this problem. (3) Treating SA as the optimization object will grant our model interesting capacity and make it cater to different requirements (see the “Optimization of different tasks” section).

### Synthetic score prediction

To directly optimize the synthetic accessibility from the generative model, we need to get feedback from the generated molecules. For this, we use SYBA to judge all sampled molecules after one epoch is finished. SYBA is capable classifying organic compounds as easy-to-synthesize (ES) or hard-to-synthesize (HS). According to SYBA, 0 serves as the threshold when estimating whether a molecule is difficult to synthesize or not. If the SYBA score is positive, the molecule is considered to be ES; otherwise, it is deemed to be HS [44].

**Fig. 5:**
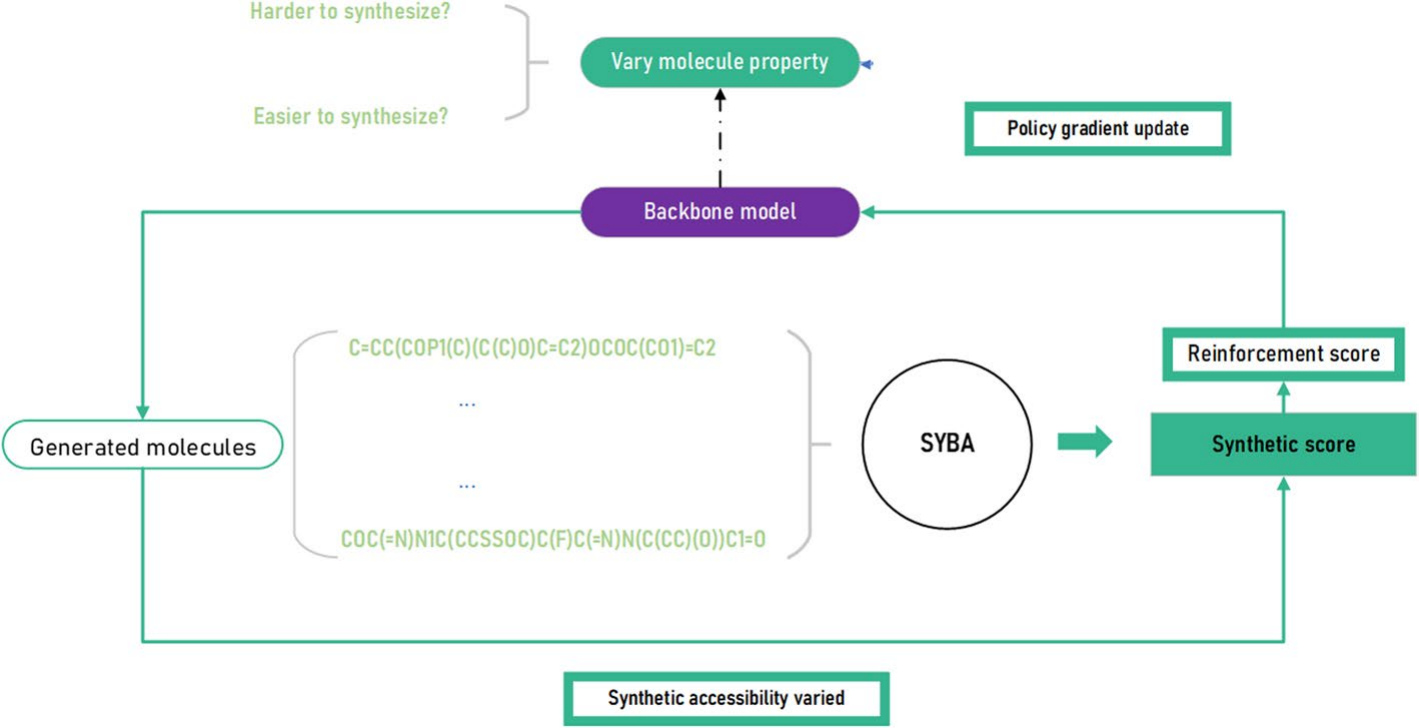
Workflow of property optimization. The generated molecules are judged by SYBA, and their attributed score is converted to reinforcement score using exponential projection. The reinforcement score is utilized by policy gradient optimization, with the molecule properties changing

**Fig. 6:**
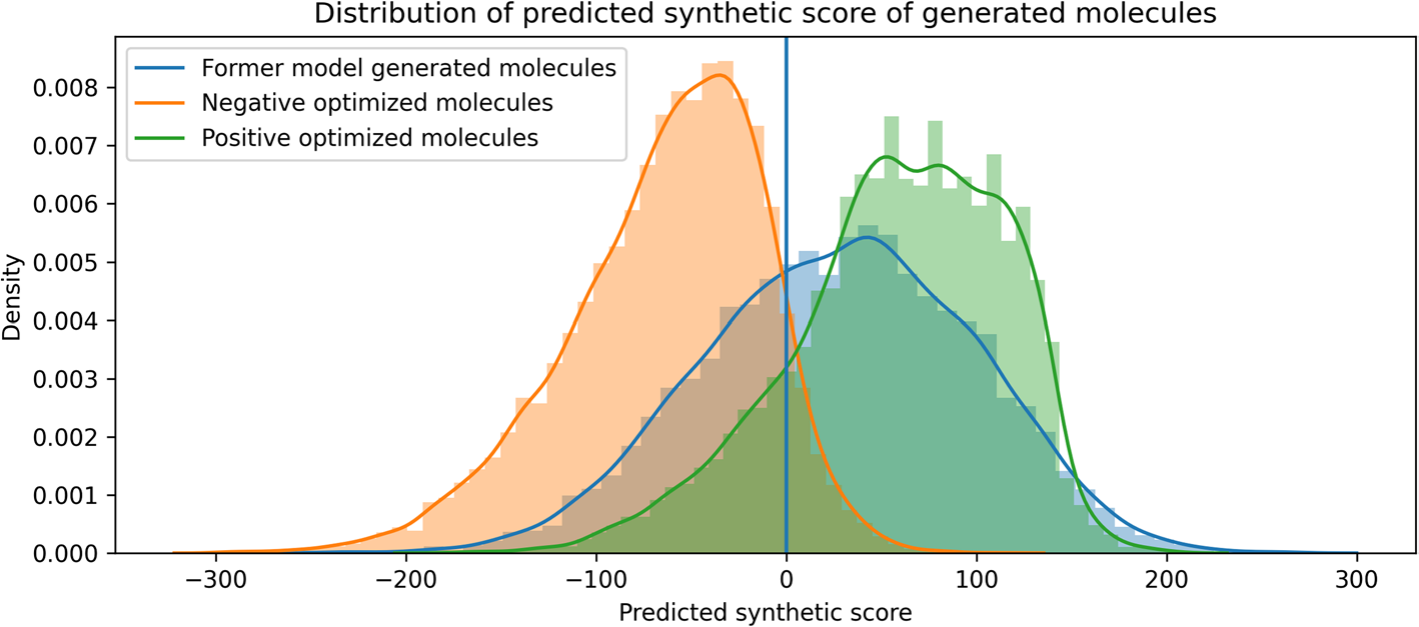
Prediction of the synthetic score by SYBA for three different models: BM, NM,and PM. The blue portion illustrates the scores derived from the original synthetic accessibility of molecules. Here, 97.2% of molecules are judged as HS after negative optimization while 84.5% are believed to be ES after positive optimization

For this work, we first generated numerous molecules from the original backbone model. We observed that approximately one-third of molecules should be estimated as hard to synthesize (Fig. 6). And the next section, we tried to focus on two opposite directions - 1. Make the generative molecules harder to synthesize. 2. Make the generative molecules easier to synthesize. The whole workflow is shown in Fig. 5.

### Optimization of different tasks

Our reinforcement learning pipeline contains two modules: an actor, and a critic. The actor takes current state $(s_T)$ and performs an action $(a_T)$ according to the environment. Meanwhile, the critic provides feedback based on $s_v$ and $a_v$ propels the actor to be optimized in the right direction.

In traditional training process, the goal of actor is to maximize the reward (equation 3). The derivative of this maximization is expressed by equation 4.

3 $$\begin{aligned} \begin{aligned} R(\Theta ) & = {} \mathbb {E}\left[ \left. r\left( s_{T}\right) \right| _{0}, \Theta \right] =\sum _{s_{T} \in S} p_{\Theta }\left( s_{T}\right) r\left( s_{T}\right) \end{aligned} \end{aligned}$$

4 $$\begin{aligned} \begin{aligned} \nabla \bar{R}_{\theta } & = \frac{1}{N} \sum _{n=1}^{N} \sum _{t=1}^{T_{n}} R\left( \tau ^{n}\right) \nabla \log p_{\theta }\left( a_{T}^{n} \mid s_{T}^{n}\right) \end{aligned} \end{aligned}$$

5 $$\begin{aligned} \begin{aligned} \nabla \bar{R}_{\theta } & = -\frac{1}{N} \sum _{n=1}^{N} \sum _{t=1}^{T_{n}} R\left( \tau ^{n}\right) \nabla \log p_{\theta }\left( a_{T}^{n} \mid s_{T}^{n}\right) \end{aligned} \end{aligned}$$

The model is trained to find a batch of parameters ($\Theta$) to maximize the reward (*R*).

In our model, the current state $s_T$ is acquired from each time step *t* according to the input token, and the action $a_T$ is provided as the output of our backbone model. During this process, we sampled a group of action pairs ($s_T$,$a_T$) from which a brand-new molecule was de novo generated. In order to maximize the mathematical expectation $\mathbb {E}$, for each reinforcement training step, we generated 10 molecules. For each molecule, we accumulate the product of reinforcement score and action pairs so that we get the reward based on equation 3 mentioned above. Following the optimized rules, we force the model to “evolve” and modify its parameters, thus making the generated molecules own higher synthetic accessibility.

However, this process is not immutable. Prior works have always maximized the mathematical expectation. In our work, we observe that this process can also be reversed; this means that, with a slight adjustment, we can reduce the reward step by step and lead the model in the opposite direction to reduce synthetic accessibility. For this process, we follow equation 5. After training, we sample 10,000 molecules from three models (Backbone Model (BM), Negative optimization Model (NM), and Positive optimization Model (PM)). The generated result of all molecules is shown in Fig. 6, where we can see a significant shift in distribution.

### Reinforcement score

According to the result of our original backbone model, the majority of synthetic score of molecules distributes from −150 to 150.

To facilitate the optimization process, an exponential projection is implemented to the original SYBA prediction, we named it as Reinforcement Score (RS) and use it as a variant of synthetic accessibility for a certain molecule following the formula $e^{\frac{1}{150}x}+e$ where *x* refers to the predicted synthesis score predicted by SYBA. Based on the converting equation, a generated molecule with a higher predicted synthetic score will also own higher reinforcement score. Before we take them into reinforcement optimization, we converted the synthetic score of all valid molecules into RS.

#### Reward truncate strategies

Reinforcement learning can be viewed as a post-processing procedure for generative models; to be specific, the reinforcement score forces the model to change toward our desired direction. However, the procedure is delicate and difficult to control, and a phenomenon called model collapse immensely affects the quality of generation. This often results in too many duplicate tokens of generated molecules and decreased performance. We view this phenomenon as caused by the stable revenue of positive examples. The model can repeat such series and get a higher reward easily. Moreover, this problem is indirect and obscure.

Some details of our task deserve further discussion:

1. We noticed that there are still parts of molecules “born” with ideal synthetic accessibility, from the optimal perspective, these molecules could waive further optimization.
2. We also noticed that our appointed reinforcement score may be too smooth for differentiating HS molecules or ES molecules. As we former mentioned, SYBA regards 0 to be the boundary of two categories. The RS projected all SYBA predictions to other continuous spaces and after that all attributed RS is positive. But the modified continuous space becomes not obvious for differentiating the two categories. For example, molecules with -10 as a predicted SA score, and after the exponential projection, its RS will be $e^{-\frac{1}{15}}+e$; and a molecule with 10 as the former, its RS will be $e^{\frac{1}{15}}+e$. We can see that the difference seems too slight after the projection and we expect to provide the model a more clear instruction when it conducts the task of positive optimization.

**Fig. 7:**
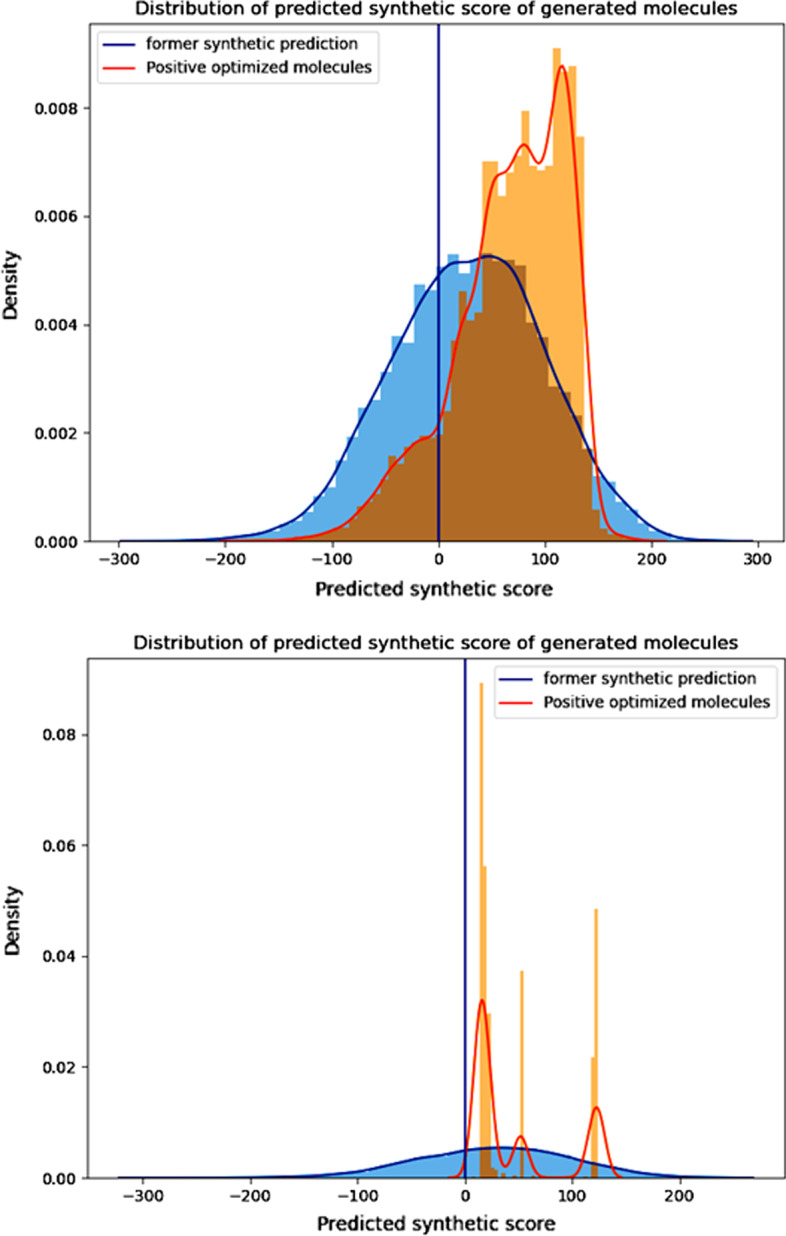
Comparison between model implemented our strategies and not after training 10 epochs. The post-processing is conducted on the same model with only utilizing the defined rules or not as the difference. For each model, we sampled 10 molecules at a single optimization timestep and repeat 20 times for a single epoch, the total epoch is set to 10 in our experiment. The upper figure shows that with proper strategies, the navigation of chemical space is feasible in comparison with the collapse model without any constraint

To solve all mentioned problems, a truncation of the reinforcement score is utilized to ensure better training results. We try to utilize the ideology of activating functions such as Relu, which exerts a non-linear transformation of the given expression. Here we first set an “optimal threshold” to exclude these molecules while conducting action-pair sampling. Any ES molecules over this threshold will not contribute to the calculation of the next step. And in our experiment, this threshold is set to 150 (before converting it to the reinforcement score). For other ES examples, only half of their reinforcement score contributes to the calculation of the next step. Actually, we try to reasonably reduce the reward of these examples to some extent thus getting rid of being dominated by token combinations with better rewards too much. To evaluate this, we did experiment with differences on adding these strategies or not, and the comparison is shown in Fig. 7. The result shows the issued strategies mitigated the phenomenon of ’model collapse’ and ensured the richness of generated molecules.

### Halfway-targeted drug-like molecules exploration

In the next part, we utilized STONED-SELFIES - an algorithm using structural evolution to quickly explore the medium molecules; Following the metrics issued in their works, the joint similarity ensures the evolution of the midbody molecule has similarities to their parents (for details, see the original paper [27]). And for our model, the light-weighted design empowers it to conduct quick exploration. Thus, we aim for using its character to search the enormous chemical space efficiently and find the ideal molecule candidate as the evolution endpoint. Hereby the two parts can be combined together for designated molecule evolution. And the detail of our experiments is listed below:

(1) Following the former parts to train the generative model with positive optimization, then we conduct molecule selection to choose the best molecules with ideal LogP and QED score (calculated by Rdkit). (2) Assign the “best” candidates as the evolution endpoint and drug candidates as the starting point to conduct structure evolution using STONED-SELFIES, see Fig. 9.

Thus, the pinpoint of our method lies in finding the ideal evolution target and the purpose is to do the structural evolution from one to another. During this process, a bunch of midbodies will be explored in the near chemical space. With proper selection strategies, we could derive molecules with both ideal properties and similarities to their parents. And we called this method - Halfway-targeted drug-like molecule exploration.

## Results

### Speed and performance

In this study, we re-trained a backbone model without inheriting the same parameter or structure of former excellent works [7, 32, 45] and adopted the SELFIES presentation. Our model demonstrates high performance throughout the whole training process with approximately 4.9 million parameters,which is only one-tenth of the backbone models of others [45]. Among our concepts, the sampling capacity matters[46], for the reason of efficiently searching for a proper evolution target, thus we take the model structure into consideration. The reduction of structures lets our model works rapidly without losing performance (BM and NM), even after the reinforcement optimization. While the model is working on a laptop with a graphic card (GTX 1060 with 6GB video memory), it still reached a high speed of generating approximately 1k novel molecules in less than a single second.

**Fig. 8:**
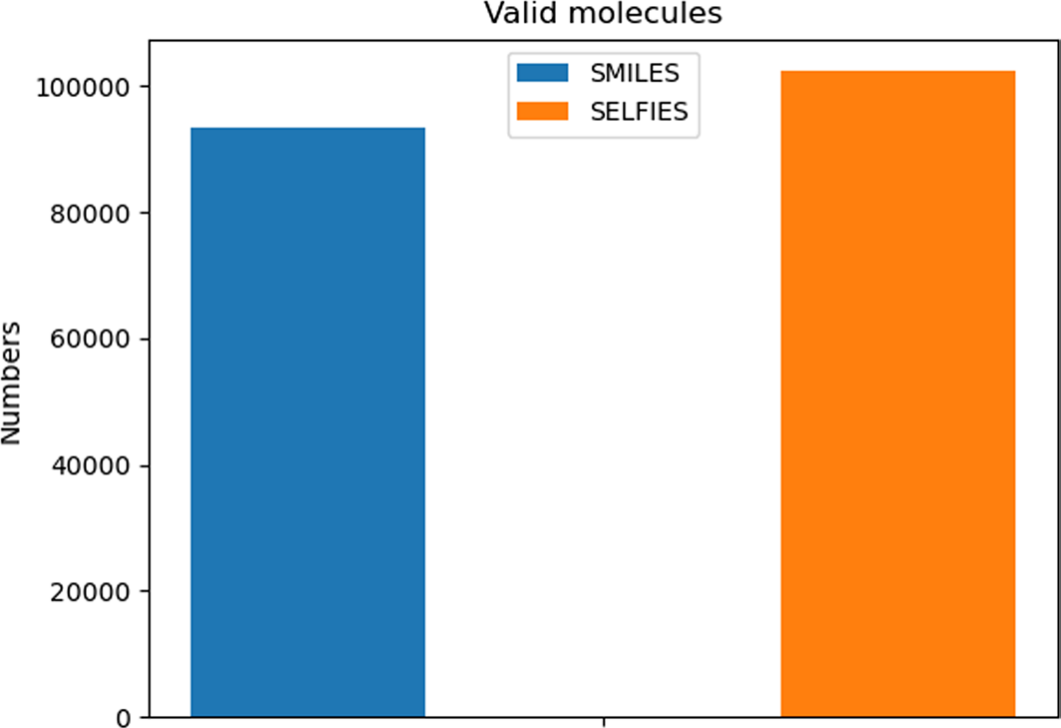
Valid molecules among all samplers. For model adopting SMILES, the valid number is 9.3k, comparing nearly 100% validity for SELFIES

And we conduct several experiments to comprehensively validate the potency of our proposed pipeline. The first is comparing the adoption of different molecule presentations (SMILES and SELFIES). During the training or sampling stage, the backbone model is trained on an NVIDIA 3090 GPU with 24 Gigabyte memory; it takes only 90 seconds to finish a single training epoch. After 10 training epochs, 100,000 molecules are randomly sampled to evaluate the differences in molecule presentations. The result is visualized in Fig. 8. Model embedding with SELFIES presentation can generate molecules with 100% validity, 99.87% uniqueness, and 99.23% for novelty; for SMILES, these values are 91.15%, 99.67%, and 98.28%, respectively. This comparison indicates that under our circumstance, adopting SELFIES presentation may be preferred for molecule generation tasks (this is because validity matters more than other metrics). From another perspective, a longer training time or increasing model parameters will make model embedding with SMILES work better; it could also be concluded that adopting SELFIES in our model makes the training procedure more efficient.

After training toward different expectations, we sampled 10,000 molecules from each model as usual and calculated some standard metrics. The result is shown in Table 1. The definitions of metrics are as follows:

6 $$\begin{aligned} \begin{aligned} Uniqueness =\frac{\left| {\text {set}}\left( V_{m}\right) \right| }{\left| V_{m}\right| } \end{aligned} \end{aligned}$$

7 $$\begin{aligned} \begin{aligned} Validity = \frac{\left| V_{m}\right| }{S_{m}} \end{aligned} \end{aligned}$$

where $S_{m}$ means a batch of sampled molecules and $V_{m}$ represents chemical valid molecules for a single batch in $S_{m}$.

**Table 1:** Performance of Magicmol in terms of different metrics

Models	Validity (%)	Uniqueness (%)	Novelty (%)	Training set	Synthesizability (%)	Druglikeness (%)	Model parameters	Match rules (%)
Magicmol (BM)	100.00	99.87	99.23	0.89 M	0.655	0.516	4.9 M	66.86
Magicmol (NM)	96.22	98.63	100.00		0.027	0.127		17.82
Magicmol (PM)	100.00	43.30	99.47		0.885	0.714		91.48
Magicmol (SMILES)	91.15	99.67	98.28		0.715	0.579		73.65

Synthesizability: generated molecules judged as ES by SYBA; Druglikeness: average QED score of generated molecules done by Rdkit; Match Rules: the percentage of sampled molecules matching Lipinski’s Rule of Five

According to the results shown in Table 1, NM holds a similar capacity for generating molecules with high diversity and validity as BM, but also has terrible drug-likeness. Here we notice a significant drop in the uniqueness score of PM: from 99.87% to 43.30%. And the possible explanation are listed below. First, the positive optimization procedure can be viewed as propelling the model to navigate from the original chemical space to our desired druggable chemical space based on SYBA feedback. Thus, ideal tokens and combinations are granted higher reinforcement scores, which explains the sequence repetition during the generation process. Second, this phenomenon could also be interpreted as PM catching drug-like features but the druggable chemical space being relatively narrow compared to the original chemical space.

To verify our these speculations, we attempt to multi-dimensionally evaluate the quality of PM-sampled molecules beyond the single number. To accomplish this, we use a filter function based on Lipinski’s Rule of Five [47] to extract more druggable molecules from all samplers. The percentage of molecules that pass the filter are identified together with the average QED, SA derived from SYBA in Table 1. The figure that matches the rules rises from 66.86% to 91.48%. compared with that of BM. Although PM faces a decrease in uniqueness, all other druglike properties are still ideal and evidently better than those of BM, which proves our hypothesis. Besides, PM is not the terminal of our pipeline. Since the concentrated distribution of druglike molecules may facilitate the subsequent workflow to some extent, we think that such a reduction is still acceptable.

### Molecule evolution

**Fig. 9:**
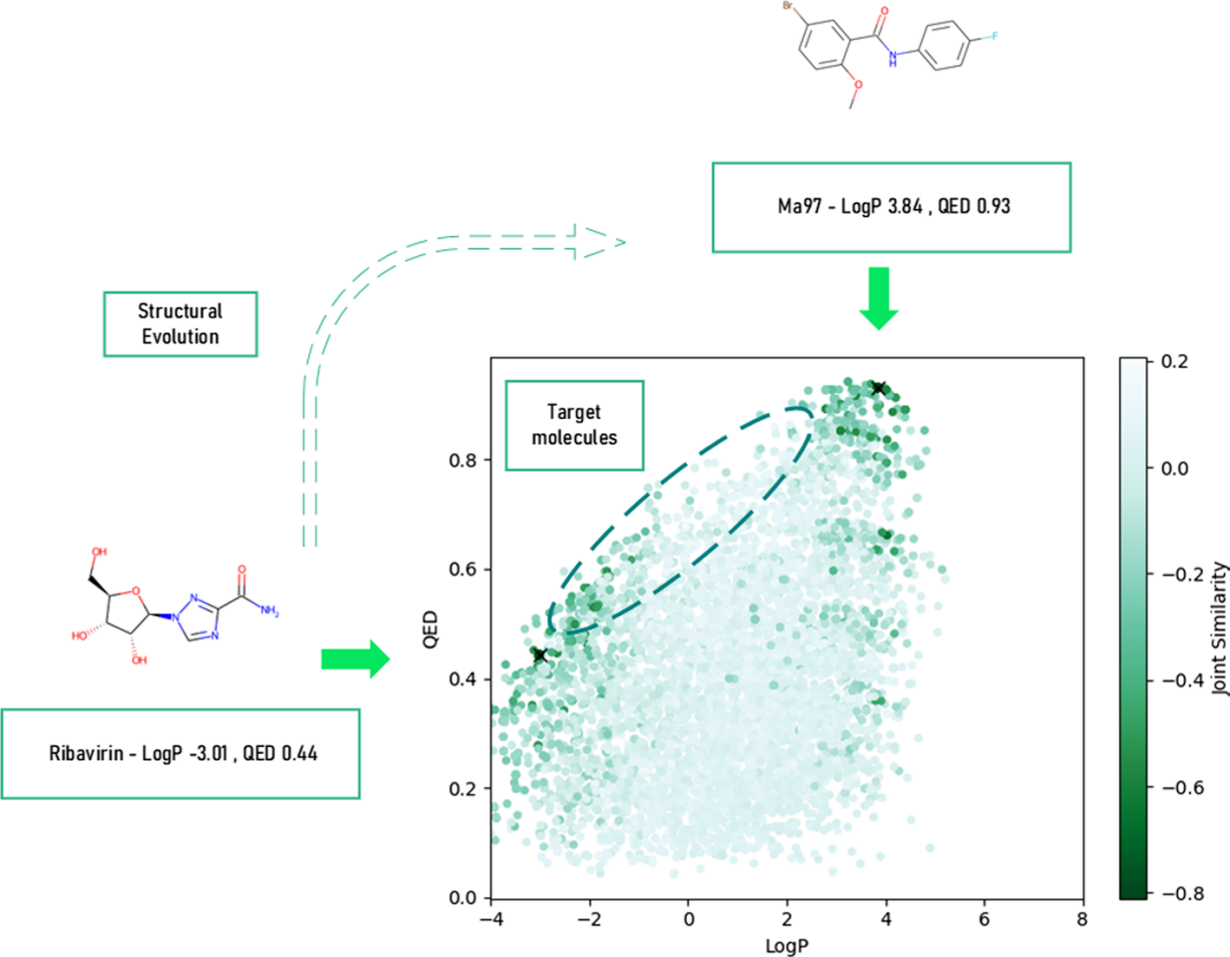
An evolution process between the formerly mentioned molecules. The molecules with better physicochemical properties are circled by the green dotted ellipse

An example of the molecule evolution process is shown in Fig. 9. The evolution starts with a drug candidate, i.e., Ribavirin (LogP $-$3.01, QED 0.44), and ends with the generated molecule Ma97 [SMILES: COC1=CC=C(Br)C=C1C(=O)NC2=CC=C(F)C=C2, LogP 3.84, QED 0.93]. During this process, STONED-SELFIES applies reasonable string manipulation. The modification of molecule presentation can be seen as a process of exploring the near chemical space around the specified molecule. Our molecule with ideal drug-like properties will lead the direction of modification (the joint similarity acts as a structural constraint). Although the evolution process indeed has some randomness, its advantage is distinct also. First, it is time-consuming and needs fewer computational resources, and thus it can be replicated round by round to extensively explore the surrounding chemical space. Second, the evolution is structurally dense because at each timestep we only permit up to two tokens to do alternation; therefore, the evolution is explainable and changes between different fragments can be detected and analyzed. Third, the whole process is done step-by-step. We first explicitly optimize the SA of generated molecules and then optimize other chemical properties; we implicitly reach the goal of multi-object optimization, which is also a dilemma in this field.

## Discussions

In this paper, we proposed Magicmol, which focuses on utilizing the advantages of two categories of methods: the exploration capacity of DGMs, and the evolution abilities of GAs. We first designed an RNN-based backbone model and conducted optimization, thus empowering molecules generation with ideal chemical structures. Then we combined these structures with STONED-SELFIES to perform molecule evolution and explore near chemical space to optimize certain molecules. In the next section, we will discuss further opinions and potential applications of Magicmol.

### De novo drug design

To be honest, Magicmol is not born for de novo devise drug-like molecules. A potent drug candidate is a combination of several aspects such as logP, QED, Absorption, Distribution, Metabolism, Excretion, and Toxicity (ADMET). We expect ideal drug-like molecules to have all these characteristics. During the reinforcement learning optimization, a single property being varied may lead to the changing of another property [7]; the solution is modifying these properties one by one, which works well but also takes time and increases computational complexity.

**Fig. 10:**
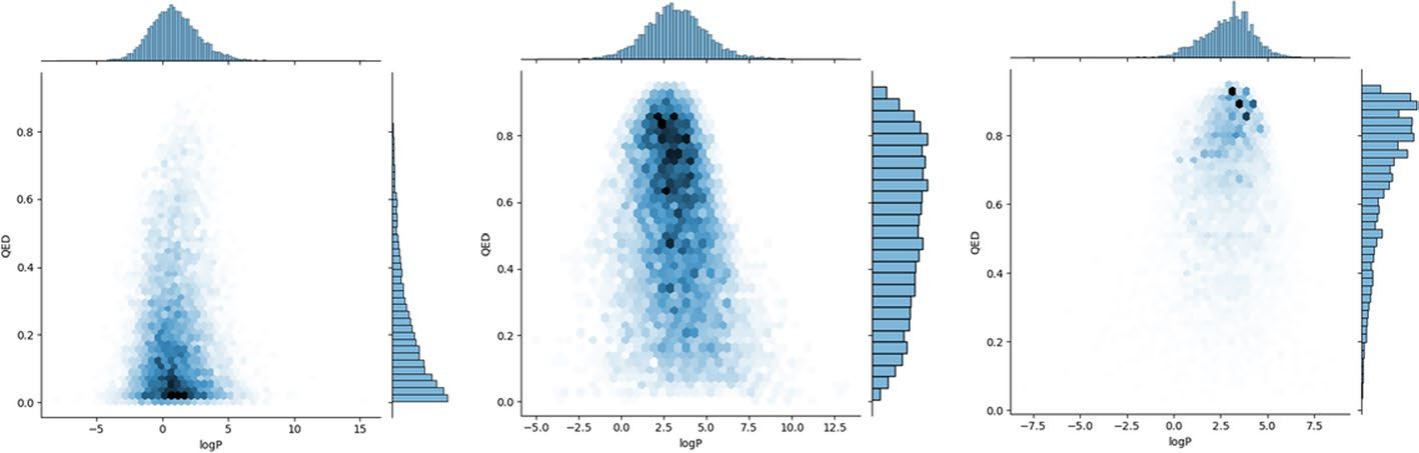
QED and logP scores of generated molecules after reinforcement learning optimization. We derived 10 batches (10,240) of molecules from our generative model. **a** NM, **b** BM, and **c** PM. QED and logP scores are acquired by Rdkit. There is a massive difference in some drug-like properties between these generated molecules, even though we just modified the synthetic accessibility

Thus, we tried to focus on altering only one significant property - synthetic accessibility, and we conducted a series of experiments to assess molecule properties after our proposed optimization. There is no absolute evidence proving that the structural complexity is binding with drug-like factors. Still, we witnessed a massive difference between these generated molecules after changing their synthetic accessibility (see Fig. 10).

Even though these properties are not perfectly positively correlated with synthetic accessibility, we still observed that, accompanying structural optimization, the drug-likeness of generated molecules increases, especially for the averaged QED score, which increases from 0.51 (BM) to 0.69 (PM). This result matches our expectation: this increase in some metrics is achieved by getting feedback and optimizing, which results in modified network parameters. Thus, the model’s capability to generate ideal molecules is enhanced. Furthermore, we think that Magicmol mitigated the difficulty of retro-synthesize route identification to some extent with the less complex generative molecules.

### Synthetic accessibility variation

We tried to reverse the rule of policy gradient so that our model can be used to vary the synthetic accessibility in a different direction. This can be utilized for either improving the synthetic accessibility of molecules or simply generating lots of hard-to-synthesize compounds without introducing any other superparameters; moreover, we eliminate the need for domain knowledge. To the best of our knowledge, other models have not emerged that try to vary the property of molecules directly from the generative model to the opposite direction.

**Fig. 11:**
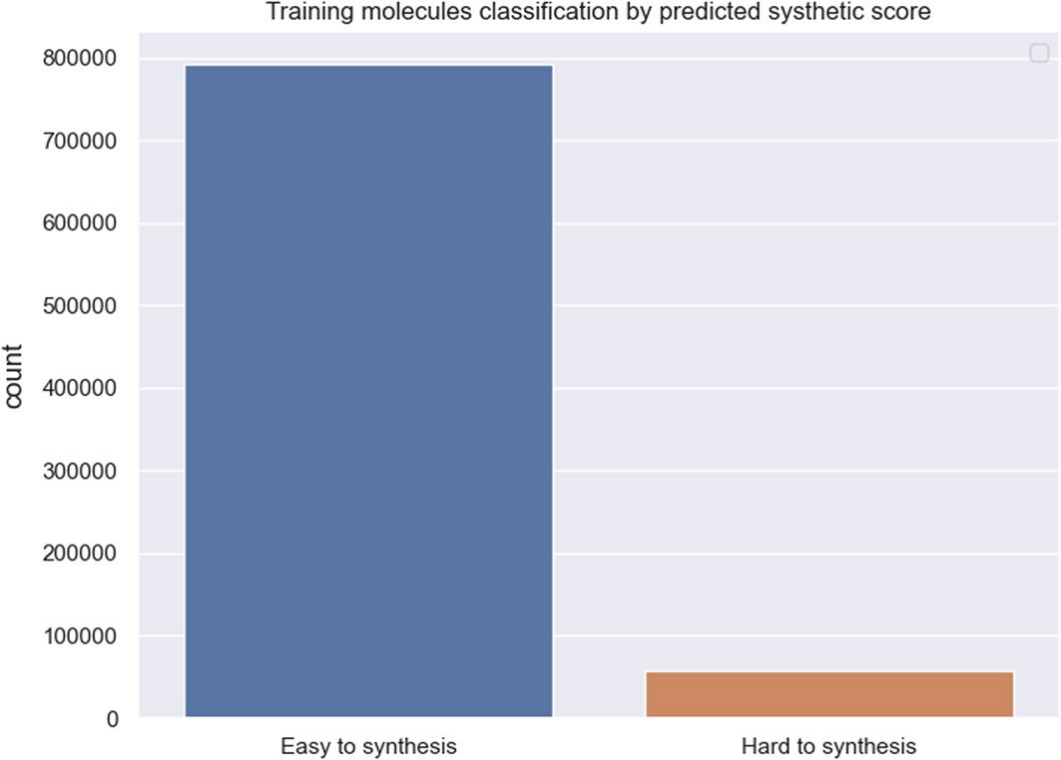
Distribution of training molecules according to their predicted synthetic score; there are approximately 13 times more positive samples than negative samples

**Fig. 12:**
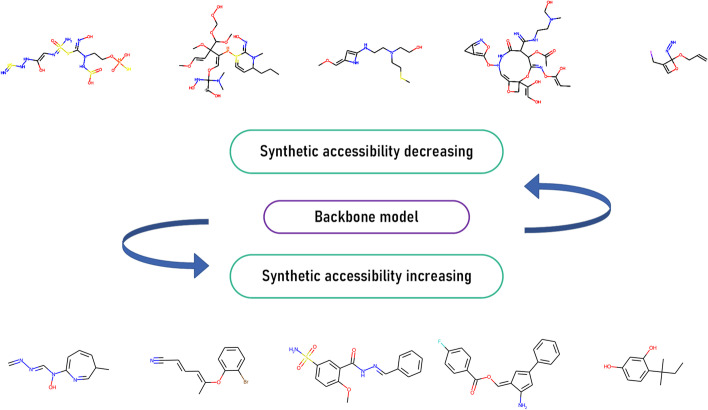
Generated molecules of different synthetic accessibility. The top line: the top five molecules generated with the highest synthetic accessibility. The bottom line: the top five molecules generated with the lowest synthetic accessibility

In the real world, intuitively, most compounds are designed to be easier to synthesize. We tried to derive the synthetic score of the original training set, and the result is shown in Fig. 11. Only approximately 7% of the compounds are judged to be difficult to synthesize. In some particular tasks, e.g., the training of SYBA, a model should balance the number of negative samples and positive samples while preparing the training set. Magicmol may serve as a high-velocity negative sample generation tool, and thus could be a solution. Figure 12 lists some generated molecules after reinforcement optimization in different directions.

## Conclusions

In this paper, we proposed Magicmol, which focused on utilizing the advantages of two categories of methods: the exploring capacity of DGMs and the evolution abilities of GAs. The idea initially seemed contradictory, but actually they can be reasonably combined. We empowered our model to generate molecules with ideal chemical structures while utilizing structural constraints that facilitate the following evolution steps. The pipeline could conduct quick exploration of enormous chemical space. Moreover, Magicmol solved the model collapse problem to some extent and provided potential solution for some problems among in silicon drug design field such as negative sampling.

## Data Availability

The source codes of our model is available at https://github.com/Josefjosda/Magicmol. The training data can be derived from https://www.ebi.ac.uk/chembl/.

## Abbreviations

BM: Backbone model
PM: Positive optimization model
NM: Negative optimization model
DGM: Deep generative model
GA: Genetic algorithm
SS: Reinforcement score
RNN: Recurrent neural network
VAE: Variational autoencoder
GAN: Generative adversarial network
SYBA: Synthetic Bayesian classifier
GRU: Gated recurrent unit
LSTM: Long short-term memory
ES: Easy-to-synthesize
HS: Hard-to-synthesize
QED: Quantitative estimate of drug-likeness
ADMET: Absorption, distribution, metabolism, excretion, toxicity
